# Crystal structure of dimethyl-1κ^2^ *C*-bis(μ-4-methylphenolato-1:2κ^2^ *O*:*O*)(*N*,*N*,*N*′,*N*′-tetramethylethylenediamine-2κ^2^ *N*,*N*′)indium(III)lithium(I)

**DOI:** 10.1107/S2056989015023476

**Published:** 2015-12-12

**Authors:** Glen G. Briand, Andreas Decken, Marshall R. Hoey

**Affiliations:** a Department of Chemistry and Biochemistry, Mount Allison University, Sackville, New Brunswick, E4L 1G8, Canada; b Department of Chemistry, University of New Brunswick, Fredericton, New Brunswick, E3B 5A3, Canada

**Keywords:** crystal structure, bimetallic, indium, lithium, phenolate, spiro­cyclic

## Abstract

The mixed bimetallic title compound, [InLi(CH_3_)_2_(C_7_H_7_O)_2_(C_6_H_16_N_2_)] or [(tmeda)Li-μ-(4-MeC_6_H_4_O)_2_InMe_2_] (tmeda is *N*,*N*,*N*′,*N*′-tetra­methyl­ethylenedi­amine), exhibits a four-membered LiO_2_In ring core *via* bridging 4-methyl­phenolate groups. The Li and In atoms are in distorted tetra­hedral N_2_O_2_ and C_2_O_2_ bonding environments, respectively. The Li atom is further chelated by a tmeda group, yielding a spiro­cyclic structure.

## Related literature   

For other bimetallic alkali–triel chalcogenolates, see: Niemeyer & Power (1997 ▸); Clegg *et al.* (1999 ▸); Muñoz *et al.* (2011 ▸, 2014 ▸); Uhl *et al.* (1994 ▸); Adonin *et al.* (2005 ▸); Soki *et al.* (2008 ▸); Normand *et al.* (2012 ▸). For metal-containing ligands, see Simmonds & Wright (2012 ▸). For organometallic precusors for indium tin oxide (ITO), see: Aksu & Driess (2009 ▸); Veith & Kunze (1991 ▸). For dimeric di­methyl­indium phenolates [Me_2_InO*R*]_2_, see: Briand *et al.* (2013 ▸, 2010 ▸); Beachley *et al.* (2003 ▸); Häusslein *et al.* (1999 ▸); Blake *et al.* (2011 ▸); Bradley *et al.* (1988 ▸); Trentler *et al.* (1997 ▸).
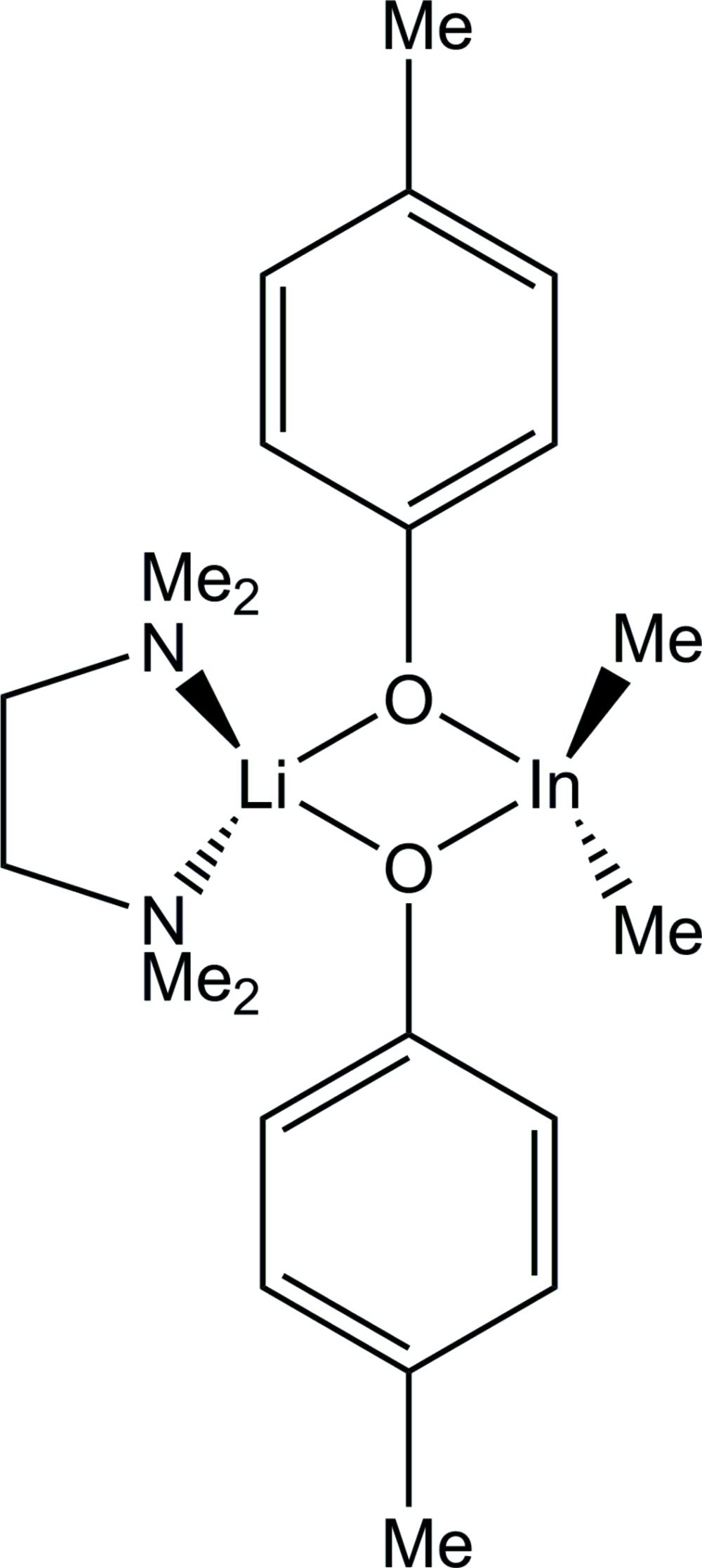


## Experimental   

### Crystal data  

[InLi(CH_3_)_2_(C_7_H_7_O)_2_(C_6_H_16_N_2_)]
*M* *_r_* = 482.29
Monoclinic, 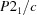

*a* = 9.0991 (8) Å
*b* = 16.4481 (15) Å
*c* = 16.4256 (15) Å
β = 91.956 (1)°
*V* = 2456.9 (4) Å^3^
*Z* = 4
Mo *K*α radiation
μ = 0.98 mm^−1^
*T* = 188 K
0.65 × 0.60 × 0.60 mm

### Data collection  

Bruker SMART1000/P4 diffractometer
Absorption correction: multi-scan (*SADABS*; Sheldrick, 2008*a* ▸) *T*_min_ = 0.569, *T*_max_ = 0.591
16648 measured reflections
5459 independent reflections
4921 reflections with *I* > 2σ(*I*)
*R*_int_ = 0.025

### Refinement  

*R*[*F* ^2^ > 2σ(*F* ^2^)] = 0.025
*wR*(*F* ^2^) = 0.067
*S* = 1.05
5459 reflections
261 parameters
H-atom parameters constrained
Δρ_max_ = 0.56 e Å^−3^
Δρ_min_ = −0.27 e Å^−3^

### 

Data collection: *SMART* (Bruker, 1999 ▸); cell refinement: *SAINT* (Bruker, 2006 ▸); data reduction: *SAINT*; program(s) used to solve structure: *SHELXS97* (Sheldrick, 2008*b* ▸); program(s) used to refine structure: *SHELXL2013* (Sheldrick, 2015 ▸); molecular graphics: *DIAMOND* (Brandenburg, 2012 ▸); software used to prepare material for publication: *SHELXTL* (Sheldrick, 2008*b* ▸).

## Supplementary Material

Crystal structure: contains datablock(s) I. DOI: 10.1107/S2056989015023476/lh5799sup1.cif

Structure factors: contains datablock(s) I. DOI: 10.1107/S2056989015023476/lh5799Isup2.hkl

Supporting information file. DOI: 10.1107/S2056989015023476/lh5799Isup4.cdx

**Fig. 1.**
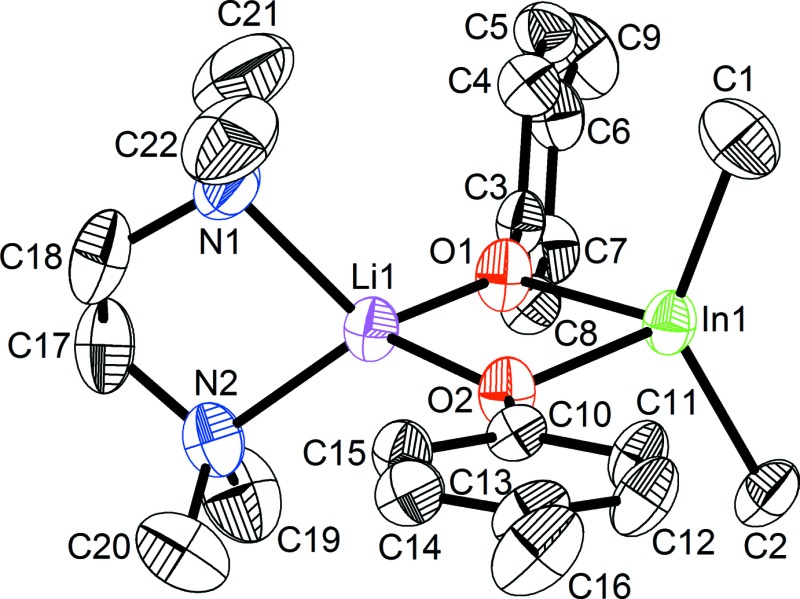
The molecular structure of (I), with displacement ellipsoids drawn at the 50% probability level. H atoms have been omitted for clarity.

I . DOI: 10.1107/S2056989015023476/lh5799fig1.tif
The mol­ecular structure of (**I**), with displacement ellipsoids drawn at the 50% probability level. H atoms have been omitted for clarity.

CCDC reference: 1440726

Additional supporting information:  crystallographic information; 3D view; checkCIF report

## supplementary crystallographic information

### S1. Comment

Ligands containing metal bridgeheads are useful for the generation of mixed metal species with novel physical properties and reactivity (Simmonds *et al.*, 2012). In our efforts to generate organometallic In/Sn precusors for indium tin oxide (ITO) semiconductor species (Aksu *et al.*, 2009; Veith *et al.*, 1991), we have isolated the Li^+^ salt of the anionic ligand [(4-MeC_6_H_4_O)_2_InMe_2_]^−^. The structure of [(tmeda)Li-µ-(4-MeC_6_H_4_O)_2_InMe_2_] (tmeda = *N*,*N*,*N*',*N*'-tetra­methyl­ethylenedi­amine) (I) (Fig. 1) exhibits a four-membered LiO_2_In ring core in which Li1 and In1 centre are bridged *via* the oxygen atoms of two 4-MeC_6_H_4_O ligands. The In—O bond distances [In1—O1 = 2.125 (1), In1—O2 = 2.141 (1) Å] are larger than the Li—O bond distances [Li1—O1 = 1.889 (4), Li1—O2 = 1.926 (3) Å] as a result of the larger covalent radius of In *versus* Li. However, the LiO_2_In ring is nearly planar [In1—O1—Li1—O2 = −4.5 (1)°]. In addition to the In—O bonds, In1 is also bonded to the carbon atoms of two methyl groups, resulting in a distorted tetra­hedral C_2_O_2_ bonding environment for indium [O1—In1—O2 = 78.32 (5), C1—In1—C2 = 133.8 (1)°]. Li1 is also bonded to two nitro­gen atoms of a chelating tmeda ligand, resulting in a distorted tetra­hedral N_2_O_2_ bonding environment for lithium [O1—Li1—O2 = 89.9 (2), N1—In1—N2 = 86.8 (2)°]. The overall result is a bimetallic spiro­cyclic arrangement. The 4-MeC_6_H_5_ rings are displaced slightly toward the Me_2_In group [C3—O1—In1 = 121.0 (1), C10—O2—In1 = 125.0 (1), C3—O1—Li1 = 142.4 (2), C10—O2—Li1 = 137.9 (2)°] and are nearly orthogonal [88.63 (6)°]. The geometries at the bridging O atoms are distorted trigonal planar [Σ*X*—O1—*X* = 360.0, Σ*X*—O2—*X* = 357.9°]. The structure resembles those of di­methyl­indium phenolates [Me_2_InO*R*]_2_, which form bimetallic species in the solid state *via* inter­molecular In—O coordinate bonding inter­actions (Briand *et al.*, 2013; Briand *et al.*, 2010; Beachley *et al.*, 2003; Häußlein *et al.*, 1999; Blake *et al.*, 2011; Bradley *et al.*, 1988; Trentler *et al.*, 1997). These structures feature distorted tetra­hedral geometries at In, distorted trigonal planar or slightly pyramidal geometries at O, and near planar In_2_O_2_ ring cores. For other bimetallic alkali-triel chalcogenolates, see: Niemeyer *et al.* (1997); Clegg *et al.* (1999); Muñoz *et al.* (2011); Uhl *et al.* (1994); Adonin *et al.* (2005); Soki *et al.* (2008); Muñoz *et al.* (2014); Normand *et al.* (2012).

### S2. Synthesis and Crystallization

Synthesis of [(tmeda)Li-µ-(4-MeC_6_H_4_O)_2_InMe_2_]. [4-MeC_6_H_4_O]Li (0.143 g, 1.25 mmol) was added to a stirrred solution of InMe_3_ (0.200 g, 1.25 mmol) in di­ethyl ether (10 ml). After1 h, 4-MeC_6_H_4_OH (0.064 g, 0.60 mmol) in di­ethyl ether (3 ml) was added. After 2 h, tmeda (0.145 g, 1.25 mmol) was added. After 1 h, the reaction mixture was filtered, and the filtrate concentrated to 5 ml and allowed to sit at 277 K. After 1 d, the solution was filtered to yield colourless crystals of I (0.188 g, 0.490 mmol, 82%). Anal. Calc. for C_22_H_36_InLiN_2_O_2_: C, 54.78; H, 7.52; N, 5.81. Found: C, 54.56; H, 8.01; N, 5.67. Mp 426–428 K. FT-Raman (cm^−1^): 127 s, 170 m, 297 w, 342 w, 502 *vs* [ν_*sym*_ (Me—In—Me)], 519 w [ν_*asym*_ (Me—In—Me)], 646 w, 766 m, 791 m, 857 m, 1155 m, 1212 w, 1288 w, 1383 w, 1438 w, 1607 w, 2841 w, 2921 m, 2959 m, 3045 w. ^1^H NMR (thf-*d*_8_, p.p.m.): 0.00 (s, 6H, *Me*_2_In), 2.31 (s, 6H, *Me*C_6_H_4_), 2.33 (s, 12H, *Me*_2_N), 2.48 (s, 4H, NC*H*_2_), 6.60 (d, ^3^J_H—H_ = 11 Hz, 4H, C_6_*H*_4_), 6.96 (d, ^3^J_H—H_ = 11 Hz, 4H, C_6_*H*_4_). ^13^C{^1^H} NMR (thf-*d*_8_, p.p.m.): −1.8 (*Me*_2_In), 19.8 (*Me*C_6_H_4_), 45.4 (*Me*_2_N), 58.2 (N*C*H_2_), 118.0 (*C*_6_H_4_), 129.5 (*C*_6_H_4_).

### S3. Refinement

H atoms were included in calculated positions and refined using a riding model.

### Crystal data

**Table:**

[InLi(CH_3_)_2_(C_7_H_7_O)_2_(C_6_H_16_N_2_)]	*F*(000) = 1000
*M**_r_* = 482.29	*D*_x_ = 1.304 Mg m^−^^3^
Monoclinic, *P*2_1_/*c*	Mo *K*α radiation, λ = 0.71073 Å
*a* = 9.0991 (8) Å	Cell parameters from 5977 reflections
*b* = 16.4481 (15) Å	θ = 2.5–28.4°
*c* = 16.4256 (15) Å	µ = 0.98 mm^−^^1^
β = 91.956 (1)°	*T* = 188 K
*V* = 2456.9 (4) Å^3^	Irregular, colourless
*Z* = 4	0.65 × 0.60 × 0.60 mm

### Data collection

**Table:**

Bruker SMART1000/P4 diffractometer	5459 independent reflections
Radiation source: fine-focus sealed tube, K760	4921 reflections with *I* > 2σ(*I*)
Graphite monochromator	*R*_int_ = 0.025
φ and ω scans	θ_max_ = 27.5°, θ_min_ = 1.8°
Absorption correction: multi-scan (*SADABS*; Sheldrick, 2008*a*)	*h* = −11→11
*T*_min_ = 0.569, *T*_max_ = 0.591	*k* = −21→21
16648 measured reflections	*l* = −21→20

### Refinement

**Table:**

Refinement on *F*^2^	Primary atom site location: structure-invariant direct methods
Least-squares matrix: full	Secondary atom site location: difference Fourier map
*R*[*F*^2^ > 2σ(*F*^2^)] = 0.025	Hydrogen site location: inferred from neighbouring sites
*wR*(*F*^2^) = 0.067	H-atom parameters constrained
*S* = 1.05	*w* = 1/[σ^2^(*F*_o_^2^) + (0.0318*P*)^2^ + 1.1193*P*] where *P* = (*F*_o_^2^ + 2*F*_c_^2^)/3
5459 reflections	(Δ/σ)_max_ = 0.002
261 parameters	Δρ_max_ = 0.56 e Å^−^^3^
0 restraints	Δρ_min_ = −0.27 e Å^−^^3^

### Special details

**Table:**

Experimental. Crystal decay was monitored by repeating the initial 50 frames at the end of the data collection and analyzing duplicate reflections
Geometry. All e.s.d.'s (except the e.s.d. in the dihedral angle between two l.s. planes) are estimated using the full covariance matrix. The cell e.s.d.'s are taken into account individually in the estimation of e.s.d.'s in distances, angles and torsion angles; correlations between e.s.d.'s in cell parameters are only used when they are defined by crystal symmetry. An approximate (isotropic) treatment of cell e.s.d.'s is used for estimating e.s.d.'s involving l.s. planes.

### Fractional atomic coordinates and isotropic or equivalent isotropic displacement parameters (Å^2^)

**Table:**

	*x*	*y*	*z*	*U*_iso_*/*U*_eq_	
In1	0.26596 (2)	0.05874 (2)	0.78772 (2)	0.03548 (6)	
O1	0.37283 (15)	0.14787 (8)	0.71713 (8)	0.0396 (3)	
O2	0.12094 (14)	0.16051 (8)	0.79780 (8)	0.0390 (3)	
Li1	0.2425 (4)	0.2341 (2)	0.7385 (2)	0.0375 (7)	
N1	0.3343 (2)	0.34149 (12)	0.79061 (12)	0.0511 (5)	
N2	0.1764 (2)	0.30590 (12)	0.63874 (11)	0.0489 (4)	
C1	0.3935 (4)	0.04698 (19)	0.89888 (16)	0.0686 (8)	
H1A	0.4979	0.0543	0.8879	0.103*	
H1B	0.3783	−0.0072	0.9220	0.103*	
H1C	0.3628	0.0884	0.9376	0.103*	
C2	0.1651 (2)	−0.02775 (13)	0.70346 (14)	0.0475 (5)	
H2A	0.0581	−0.0208	0.7024	0.071*	
H2B	0.1899	−0.0831	0.7211	0.071*	
H2C	0.2016	−0.0185	0.6488	0.071*	
C3	0.4953 (2)	0.12953 (11)	0.67653 (11)	0.0333 (4)	
C4	0.6347 (2)	0.13756 (12)	0.71342 (11)	0.0382 (4)	
H4	0.6443	0.1558	0.7682	0.046*	
C5	0.7597 (2)	0.11910 (13)	0.67083 (12)	0.0408 (4)	
H5	0.8537	0.1252	0.6971	0.049*	
C6	0.7502 (2)	0.09210 (13)	0.59107 (12)	0.0415 (4)	
C7	0.6121 (2)	0.08379 (13)	0.55467 (12)	0.0426 (5)	
H7	0.6032	0.0653	0.4999	0.051*	
C8	0.4854 (2)	0.10193 (12)	0.59630 (12)	0.0384 (4)	
H8	0.3917	0.0955	0.5699	0.046*	
C9	0.8866 (3)	0.06967 (18)	0.54574 (16)	0.0625 (7)	
H9A	0.9076	0.0117	0.5534	0.094*	
H9B	0.9702	0.1018	0.5669	0.094*	
H9C	0.8702	0.0810	0.4876	0.094*	
C10	0.0086 (2)	0.16495 (12)	0.84883 (11)	0.0334 (4)	
C11	−0.0424 (2)	0.09752 (13)	0.89039 (13)	0.0415 (4)	
H11	0.0019	0.0460	0.8824	0.050*	
C12	−0.1578 (3)	0.10467 (14)	0.94352 (14)	0.0502 (5)	
H12	−0.1910	0.0575	0.9708	0.060*	
C13	−0.2252 (2)	0.17834 (15)	0.95769 (13)	0.0471 (5)	
C14	−0.1765 (2)	0.24514 (14)	0.91485 (13)	0.0458 (5)	
H14	−0.2227	0.2963	0.9220	0.055*	
C15	−0.0617 (2)	0.23886 (13)	0.86159 (12)	0.0407 (4)	
H15	−0.0305	0.2859	0.8333	0.049*	
C16	−0.3460 (3)	0.18714 (19)	1.01892 (18)	0.0705 (8)	
H16A	−0.4306	0.2151	0.9931	0.106*	
H16B	−0.3090	0.2188	1.0658	0.106*	
H16C	−0.3762	0.1331	1.0372	0.106*	
C17	0.2618 (4)	0.37967 (17)	0.64916 (18)	0.0716 (8)	
H17A	0.2149	0.4236	0.6164	0.086*	
H17B	0.3615	0.3706	0.6285	0.086*	
C18	0.2747 (4)	0.40580 (16)	0.7358 (2)	0.0719 (8)	
H18A	0.3396	0.4540	0.7399	0.086*	
H18B	0.1764	0.4221	0.7540	0.086*	
C19	0.2068 (4)	0.2688 (2)	0.55914 (15)	0.0742 (8)	
H19A	0.1775	0.3065	0.5154	0.111*	
H19B	0.1509	0.2181	0.5528	0.111*	
H19C	0.3121	0.2571	0.5565	0.111*	
C20	0.0174 (3)	0.3218 (2)	0.64082 (18)	0.0748 (8)	
H20A	−0.0056	0.3462	0.6933	0.112*	
H20B	−0.0365	0.2705	0.6339	0.112*	
H20C	−0.0115	0.3592	0.5967	0.112*	
C21	0.4918 (3)	0.3336 (2)	0.7846 (3)	0.1069 (14)	
H21A	0.5149	0.3171	0.7291	0.160*	
H21B	0.5284	0.2924	0.8233	0.160*	
H21C	0.5389	0.3859	0.7972	0.160*	
C22	0.2953 (4)	0.3605 (2)	0.87428 (18)	0.0885 (10)	
H22A	0.3377	0.3194	0.9114	0.133*	
H22B	0.1881	0.3606	0.8781	0.133*	
H22C	0.3341	0.4142	0.8894	0.133*	

### Atomic displacement parameters (Å^2^)

**Table:**

	*U*^11^	*U*^22^	*U*^33^	*U*^12^	*U*^13^	*U*^23^
In1	0.03663 (9)	0.03030 (8)	0.04007 (8)	−0.00028 (5)	0.00951 (6)	0.00208 (5)
O1	0.0382 (7)	0.0336 (7)	0.0480 (7)	0.0027 (6)	0.0178 (6)	0.0033 (6)
O2	0.0386 (7)	0.0350 (7)	0.0443 (7)	0.0027 (6)	0.0159 (6)	0.0039 (6)
Li1	0.0400 (17)	0.0319 (16)	0.0410 (16)	−0.0002 (13)	0.0065 (13)	0.0024 (13)
N1	0.0498 (11)	0.0399 (10)	0.0635 (12)	−0.0054 (8)	0.0004 (9)	−0.0099 (9)
N2	0.0563 (11)	0.0452 (10)	0.0454 (9)	0.0051 (9)	0.0050 (8)	0.0086 (8)
C1	0.0794 (19)	0.079 (2)	0.0467 (13)	0.0079 (15)	−0.0064 (13)	0.0139 (13)
C2	0.0454 (12)	0.0363 (11)	0.0613 (13)	−0.0092 (9)	0.0056 (10)	−0.0113 (10)
C3	0.0357 (9)	0.0257 (9)	0.0390 (9)	0.0004 (7)	0.0109 (7)	0.0026 (7)
C4	0.0403 (10)	0.0406 (10)	0.0338 (9)	0.0028 (8)	0.0047 (8)	−0.0026 (8)
C5	0.0340 (10)	0.0437 (11)	0.0449 (10)	0.0035 (8)	0.0025 (8)	0.0018 (9)
C6	0.0427 (11)	0.0387 (11)	0.0441 (10)	0.0097 (9)	0.0145 (9)	0.0038 (9)
C7	0.0524 (12)	0.0421 (11)	0.0337 (9)	0.0043 (9)	0.0078 (8)	−0.0047 (8)
C8	0.0375 (10)	0.0367 (10)	0.0411 (9)	−0.0021 (8)	0.0027 (8)	−0.0021 (8)
C9	0.0531 (14)	0.0774 (18)	0.0584 (14)	0.0221 (13)	0.0217 (12)	0.0033 (13)
C10	0.0319 (9)	0.0356 (10)	0.0329 (8)	−0.0025 (7)	0.0048 (7)	−0.0049 (7)
C11	0.0453 (11)	0.0326 (10)	0.0475 (10)	−0.0047 (8)	0.0158 (9)	−0.0079 (9)
C12	0.0567 (13)	0.0429 (12)	0.0525 (12)	−0.0154 (10)	0.0244 (10)	−0.0091 (10)
C13	0.0376 (10)	0.0536 (13)	0.0512 (11)	−0.0087 (9)	0.0145 (9)	−0.0189 (10)
C14	0.0381 (10)	0.0452 (12)	0.0546 (12)	0.0059 (9)	0.0073 (9)	−0.0123 (10)
C15	0.0388 (10)	0.0376 (10)	0.0461 (10)	0.0028 (8)	0.0073 (8)	−0.0001 (9)
C16	0.0580 (15)	0.0729 (18)	0.0832 (18)	−0.0151 (13)	0.0398 (14)	−0.0275 (15)
C17	0.093 (2)	0.0459 (14)	0.0762 (18)	−0.0046 (14)	0.0121 (16)	0.0229 (13)
C18	0.092 (2)	0.0334 (12)	0.091 (2)	−0.0066 (13)	0.0130 (17)	−0.0044 (13)
C19	0.100 (2)	0.077 (2)	0.0470 (13)	0.0161 (17)	0.0141 (14)	0.0074 (13)
C20	0.0626 (16)	0.100 (2)	0.0619 (15)	0.0196 (16)	−0.0017 (13)	0.0061 (16)
C21	0.0563 (18)	0.080 (2)	0.184 (4)	−0.0067 (16)	−0.005 (2)	−0.043 (3)
C22	0.117 (3)	0.085 (2)	0.0633 (17)	−0.023 (2)	−0.0025 (17)	−0.0287 (17)

### Geometric parameters (Å, º)

**Table:**

In1—O1	2.1252 (13)	C9—H9B	0.9800
In1—C1	2.138 (3)	C9—H9C	0.9800
In1—O2	2.1414 (13)	C10—C11	1.391 (3)
In1—C2	2.167 (2)	C10—C15	1.393 (3)
O1—C3	1.352 (2)	C11—C12	1.393 (3)
O1—Li1	1.889 (4)	C11—H11	0.9500
O2—C10	1.346 (2)	C12—C13	1.382 (3)
O2—Li1	1.926 (3)	C12—H12	0.9500
Li1—N2	2.092 (4)	C13—C14	1.386 (3)
Li1—N1	2.122 (4)	C13—C16	1.522 (3)
N1—C21	1.446 (4)	C14—C15	1.389 (3)
N1—C22	1.465 (4)	C14—H14	0.9500
N1—C18	1.480 (4)	C15—H15	0.9500
N2—C17	1.448 (3)	C16—H16A	0.9800
N2—C20	1.471 (3)	C16—H16B	0.9800
N2—C19	1.478 (3)	C16—H16C	0.9800
C1—H1A	0.9800	C17—C18	1.487 (4)
C1—H1B	0.9800	C17—H17A	0.9900
C1—H1C	0.9800	C17—H17B	0.9900
C2—H2A	0.9800	C18—H18A	0.9900
C2—H2B	0.9800	C18—H18B	0.9900
C2—H2C	0.9800	C19—H19A	0.9800
C3—C4	1.393 (3)	C19—H19B	0.9800
C3—C8	1.394 (3)	C19—H19C	0.9800
C4—C5	1.389 (3)	C20—H20A	0.9800
C4—H4	0.9500	C20—H20B	0.9800
C5—C6	1.383 (3)	C20—H20C	0.9800
C5—H5	0.9500	C21—H21A	0.9800
C6—C7	1.380 (3)	C21—H21B	0.9800
C6—C9	1.514 (3)	C21—H21C	0.9800
C7—C8	1.392 (3)	C22—H22A	0.9800
C7—H7	0.9500	C22—H22B	0.9800
C8—H8	0.9500	C22—H22C	0.9800
C9—H9A	0.9800		
			
O1—In1—C1	106.45 (10)	C6—C9—H9C	109.5
O1—In1—O2	78.32 (5)	H9A—C9—H9C	109.5
C1—In1—O2	108.82 (9)	H9B—C9—H9C	109.5
O1—In1—C2	107.23 (7)	O2—C10—C11	122.42 (17)
C1—In1—C2	133.76 (11)	O2—C10—C15	120.24 (17)
O2—In1—C2	108.30 (8)	C11—C10—C15	117.34 (17)
C3—O1—Li1	142.41 (15)	C10—C11—C12	120.8 (2)
C3—O1—In1	121.02 (11)	C10—C11—H11	119.6
Li1—O1—In1	96.57 (11)	C12—C11—H11	119.6
C10—O2—Li1	137.92 (16)	C13—C12—C11	121.9 (2)
C10—O2—In1	125.00 (12)	C13—C12—H12	119.1
Li1—O2—In1	94.94 (11)	C11—C12—H12	119.1
O1—Li1—O2	89.85 (15)	C12—C13—C14	117.28 (18)
O1—Li1—N2	116.35 (17)	C12—C13—C16	122.0 (2)
O2—Li1—N2	126.61 (19)	C14—C13—C16	120.7 (2)
O1—Li1—N1	117.30 (18)	C13—C14—C15	121.4 (2)
O2—Li1—N1	122.97 (18)	C13—C14—H14	119.3
N2—Li1—N1	86.82 (15)	C15—C14—H14	119.3
C21—N1—C22	110.9 (3)	C14—C15—C10	121.3 (2)
C21—N1—C18	111.5 (3)	C14—C15—H15	119.4
C22—N1—C18	108.8 (2)	C10—C15—H15	119.4
C21—N1—Li1	105.9 (2)	C13—C16—H16A	109.5
C22—N1—Li1	116.8 (2)	C13—C16—H16B	109.5
C18—N1—Li1	102.62 (18)	H16A—C16—H16B	109.5
C17—N2—C20	111.9 (2)	C13—C16—H16C	109.5
C17—N2—C19	109.5 (2)	H16A—C16—H16C	109.5
C20—N2—C19	107.9 (2)	H16B—C16—H16C	109.5
C17—N2—Li1	103.99 (18)	N2—C17—C18	112.3 (2)
C20—N2—Li1	109.79 (18)	N2—C17—H17A	109.1
C19—N2—Li1	113.73 (18)	C18—C17—H17A	109.1
In1—C1—H1A	109.5	N2—C17—H17B	109.1
In1—C1—H1B	109.5	C18—C17—H17B	109.1
H1A—C1—H1B	109.5	H17A—C17—H17B	107.9
In1—C1—H1C	109.5	N1—C18—C17	113.0 (2)
H1A—C1—H1C	109.5	N1—C18—H18A	109.0
H1B—C1—H1C	109.5	C17—C18—H18A	109.0
In1—C2—H2A	109.5	N1—C18—H18B	109.0
In1—C2—H2B	109.5	C17—C18—H18B	109.0
H2A—C2—H2B	109.5	H18A—C18—H18B	107.8
In1—C2—H2C	109.5	N2—C19—H19A	109.5
H2A—C2—H2C	109.5	N2—C19—H19B	109.5
H2B—C2—H2C	109.5	H19A—C19—H19B	109.5
O1—C3—C4	121.17 (17)	N2—C19—H19C	109.5
O1—C3—C8	120.74 (17)	H19A—C19—H19C	109.5
C4—C3—C8	118.09 (17)	H19B—C19—H19C	109.5
C5—C4—C3	120.61 (17)	N2—C20—H20A	109.5
C5—C4—H4	119.7	N2—C20—H20B	109.5
C3—C4—H4	119.7	H20A—C20—H20B	109.5
C6—C5—C4	121.43 (19)	N2—C20—H20C	109.5
C6—C5—H5	119.3	H20A—C20—H20C	109.5
C4—C5—H5	119.3	H20B—C20—H20C	109.5
C7—C6—C5	117.92 (18)	N1—C21—H21A	109.5
C7—C6—C9	120.8 (2)	N1—C21—H21B	109.5
C5—C6—C9	121.2 (2)	H21A—C21—H21B	109.5
C6—C7—C8	121.53 (18)	N1—C21—H21C	109.5
C6—C7—H7	119.2	H21A—C21—H21C	109.5
C8—C7—H7	119.2	H21B—C21—H21C	109.5
C7—C8—C3	120.41 (19)	N1—C22—H22A	109.5
C7—C8—H8	119.8	N1—C22—H22B	109.5
C3—C8—H8	119.8	H22A—C22—H22B	109.5
C6—C9—H9A	109.5	N1—C22—H22C	109.5
C6—C9—H9B	109.5	H22A—C22—H22C	109.5
H9A—C9—H9B	109.5	H22B—C22—H22C	109.5
