# Supplementary figures and images for: Crystal structure of dimethyl-1κ2 C-bis(μ-4-methylphenolato-1:2κ2 O:O)(N,N,N′,N′-tetramethylethylenediamine-2κ2 N,N′)indium(III)lithium(I)
Source: Acta Crystallogr E Crystallogr Commun. 2015 Dec 12;71(Pt 12):m257–8. doi: 10.1107/S2056989015023476 (PMC4719862; doi:10.1107/S2056989015023476)

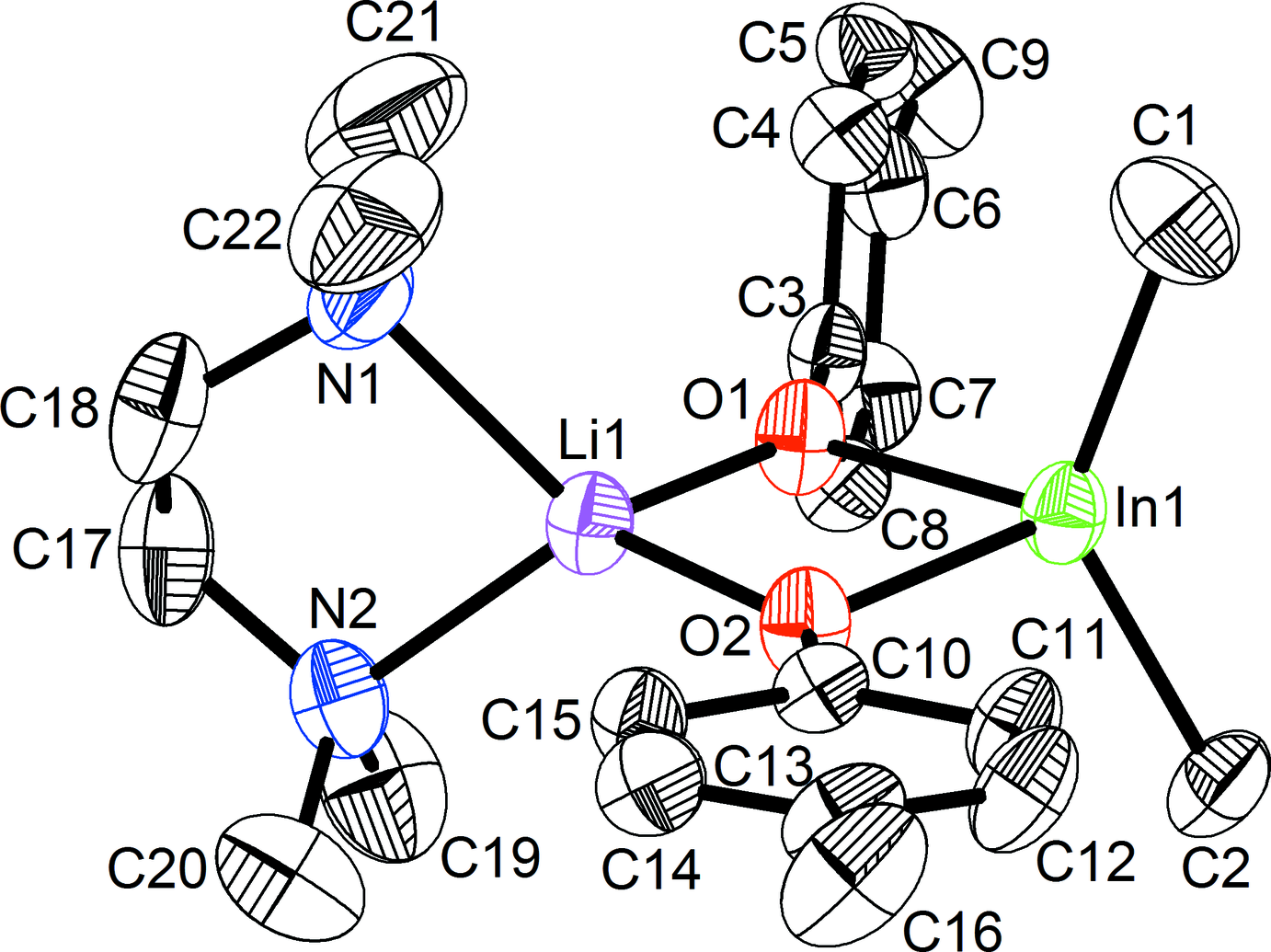

Supplement: Supplementary file 4 [file e-71-0m257-fig1.tif]
